# A UWB/Improved PDR Integration Algorithm Applied to Dynamic Indoor Positioning for Pedestrians

**DOI:** 10.3390/s17092065

**Published:** 2017-09-08

**Authors:** Pengzhan Chen, Ye Kuang, Xiaoyue Chen

**Affiliations:** School of Electrical Engineering and Automation, East China Jiaotong University, Nanchang 330013, China; pzchen@ecjtu.jx.cn (P.C.); 18252714891@163.com (Y.K.)

**Keywords:** inertial navigation, UWB, indoor positioning, symmetrical features, error correction

## Abstract

Inertial sensors are widely used in various applications, such as human motion monitoring and pedestrian positioning. However, inertial sensors cannot accurately define the process of human movement, a limitation that causes data drift in the process of human body positioning, thus seriously affecting positioning accuracy and stability. The traditional pedestrian dead-reckoning algorithm, which is based on a single inertial measurement unit, can suppress the data drift, but fails to accurately calculate the number of walking steps and heading value, thus it cannot meet the application requirements. This study proposes an indoor dynamic positioning method with an error self-correcting function based on the symmetrical characteristics of human motion to obtain the definition basis of human motion process quickly and to solve the abovementioned problems. On the basis of this proposed method, an ultra-wide band (UWB) method is introduced. An unscented Kalman filter is applied to fuse inertial sensors and UWB data, inertial positioning is applied to compensation for the defects of susceptibility to UWB signal obstacles, and UWB positioning is used to overcome the error accumulation of inertial positioning. The above method can improve both the positioning accuracy and the response of the positioning results. Finally, this study designs an indoor positioning test system to test the static and dynamic performances of the proposed indoor positioning method. Results show that the positioning system both has high accuracy and good real-time performance.

## 1. Introduction

In today’s fast-paced modern society, people are increasingly dependent on the convenience of location-based services, which are based on the precise positioning method. According to statistics, 80–90% of human activities occur indoors; thus, without interfering on the premise of privacy, the indoor location information of crowds has high commercial value.

At present, the mainstream indoor positioning technologies are ultrasound, infrared, Bluetooth, ZigBee, radio frequency identification technology (RFID), and WIFI [1,2,3,4,5,6]. This technologies meet the requirements of some indoor activities to some extent, but some shortcomings still exist, such as high cost and poor positioning accuracy. An inertial-sensor-based method, named pedestrian dead-reckoning (PDR) positioning, has the advantages of low cost, small volume, and strong autonomy, but its inertial positioning method has poor positioning accuracy and the positioning error will accumulate over time. Therefore, determining how to reduce the cumulative error of the inertial sensors is an urgent concern. Zhuang et al. [7] used the zero velocity update (ZUPT) algorithm to clear the cumulative error in each step interval, and improve the accuracy of inertial navigation to a certain extent. The error can be controlled within 2 m. The positioning accuracy of the meter level can barely meet the requirements because the conditions become more severe. Therefore, inertial positioning is usually combined with other positioning methods. Xin et al. [8] used various methods to fuse the Bluetooth tag and PDR positioning data; after experimental verification, the extended Kalman filter (EKF) fusion could achieve increased positioning accuracy and an average accuracy of less than 1 m. A disadvantage is that the cost of placement and maintenance of Bluetooth labels is high, and, Bluetooth positioning and PDR method can barely achieve precise positioning. Thus, integrating the two methods can only reach the positioning accuracy at the meter level. Ruiz et al. [9] proposed a new INS/RFID tightly-coupled indoor location method based on Kalman filter (KF), and applied ZUPT and zero angular-rate updates (ZARU) to detect pedestrian gait interval. The method can effectively eliminate the error drift and control the average positioning accuracy in 1.5 m.

The combination of positioning technologies and inertial positioning can only improve the positioning accuracy to a very small extent because the positioning accuracy of Bluetooth and RFID methods is poor. Meanwhile, UWB is not sensitive to channel fading, has low transmission signal power spectral density, low interception capacity, and low system complexity, and can provide centimeter-level accuracy, but its susceptible to blocking, which causes low dynamic performance. Pittet et al. [10] used EKF to fuse UWB/PDR data. However, EKF could not achieve good results, and even cause the filtering divergence of the nonlinear system. In addition, the KF could not correct the heading error, thereby increasing the heading error with time. Zihajehzadeh et al. [11] used two cascaded Kalman filter to fuse the UWB/IMU data and reduce the heading errors to a certain extent, but the heading angle needed 20 s to be converged; thus, the method failed to meet the dynamic response requirements. He et al. [12] used distance data from the UWB module placed on the mobile node to correct the positioning error of dead reckoning (DR) and were able to achieve an average positioning accuracy of 0.2646 m.

The aforementioned studies show that the combined positioning method of UWB/PDR can obtain high positioning accuracy and meet the requirements of most indoor environments. However, the above method neglects the dynamic performance of indoor positioning system.

Given the increasingly important role of indoor positioning plays in virtual reality (VR) and robots, dynamic performance has become an indispensable in evaluating a qualified indoor positioning system. Dynamic positioning accuracy will directly affect the user’s gaming experience, especially in VR theme parks and other indoor positioning applications. In a complex indoor environment, the independent UWB positioning can barely obtain continuous ranging information, and the autonomy and real-time performance of inertial sensors can favorably improve the dynamic performance of the integrated positioning system. However, the traditional PDR algorithm based on a single IMU cannot provide accurate positioning data. Hence, integrating UWB/PDR data directly failed to achieve the ideal static and dynamic positioning accuracy.

Inertial sensing is widely used in human motion monitoring and location positioning of various applications. However, because inertial sensors cannot accurately define the process of human movement, data drift occurs in the human positioning process, which seriously affects positioning accuracy and stability. At present, many scholars have adopted various methods based on inertial sensing to improve the performance of human motion. Although the static performance of positioning data can be improved, but the dynamic performance of positioning results still fails to meet the application requirements.

Present studies have neglected the following rules: in the process of human body movement, in order to maintain body balance, the movements of symmetrical parts have a certain degree of similarity and symmetry. Moreover, throughout the movement, a certain regularity exists between the movement of symmetrical joint bones and the movement of joint bones is similar; only a certain phase difference occurs in time. These rules can be used to accurately define the process of human motion and improve the traditional PDR algorithm.

## 2. Positioning Strategy of Distributed Inertial Sensors

PDR algorithm is a pedestrian trajectory estimation method based on inertial sensors. The traditional PDR algorithm takes the data of single-foot inertial sensor as the input and completes the pedestrian calculation through the steps of step detection, step length calculation, and heading calculation. However, the data acquisition and calculation process of a single foot causes a large error and lacks a review mechanism. The improved PDR algorithm proposed in this study is a positioning method based on the inertial data of the right and left feet. To verify the feasibility of the method, the output inertial data of the pedestrian's feet are analyzed, and the action characteristics of the human body are obtained before improving the traditional method.

### 2.1. Analysis of Human Body Movement

The motion of the human body presents periodicity during walking, which can be seen through observation. A complete gait cycle starts at the point when the heel of one foot hits the floor and ends at the point when the heel of the same foot hits the floor again. During this period, the lower limb experiences both stationary and swinging phases. The swing phase can be divided into three stages, namely, toe-off (TO), mid-stance (MS) and the heelstrike (HS). Thus, a complete gait cycle can be divided into four processes, namely, TO, MS, HS and foot flat (FF).

The movements of the lower limbs are usually symmetrical. Two inertial sensors are placed on both feet in order to verify the symmetry of the lower limb. The angular velocity curve of the Y-axis can reflect the gait cycle and the symmetry of the feet when walking by analyzing triaxial acceleration and angular velocity data.

**Figure 1:**
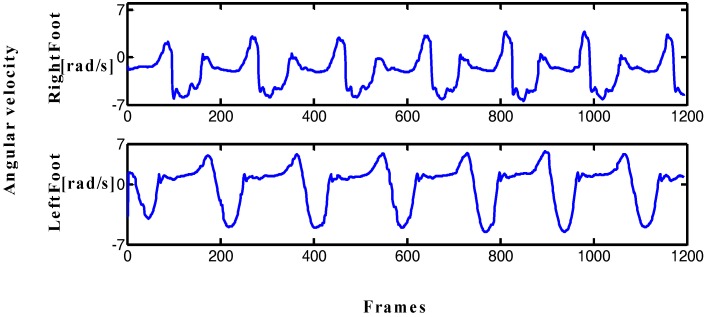
Angular velocity of both feet (1 frame = 0.017 s).

**Figure 2:**
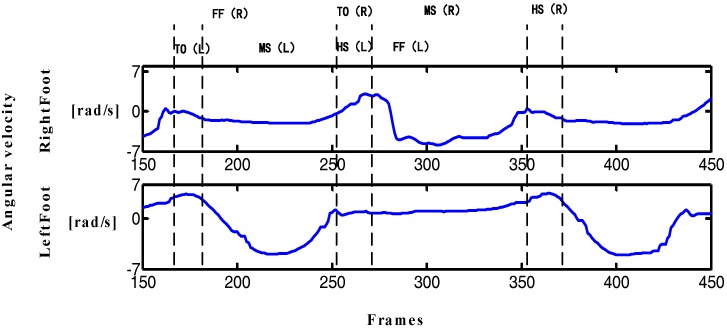
Gait cycle of both feet (1 frame = 0.017 s).

In Figure 1, the periodic characteristic of the angular velocity curve of the Y-axis is more obvious, the output curve is smooth, and the features are easy to be extract. Figure 2 shows that the gait cycle of the left and right feet alternates with each other, and the still period of the left (right) foot corresponds to the swing period of the right (left) foot. These characteristics provide the possibility of implementing the PDR algorithm and improving dynamic performance.

The traditional PDR algorithm is based on the output data of a single-foot inertial sensor. However, the reliability of single-foot data is low, leading to a large deviation in the pedestrian position estimation. Step detection method based on one foot is not accurate and lacks an inspection mechanism, especially in the step detection of PDR algorithm, thus affecting the final positioning results. Figure 2 shows that the symmetry of the human lower limb in motion can favorably provide the precision of step detection. The data in the figure and daily experience show that feet cannot be in a swing state at the same time during walking; when one foot is in the swing phase, the other foot must be in a quiescent period to maintain the stability of the body's center of gravity. This phenomenon indicates that the body is in the stationary state when two feet are stationary at the same time. According to the above characteristics, this study improves the step detection and heading estimation methods in traditional PDR algorithm by proposing a step detection method based on limb symmetry and a dynamic threshold and a heading estimation method based on angular velocity threshold. These methods can improve the precision of step detection and heading calculation and finally improve the positioning accuracy and dynamic performance of the PDR algorithm.

### 2.2. Improved PDR Indoor Positioning Algorithm

INS and PDR algorithm are two commonly used methods in navigation [13]. The advantages of the PDR algorithm is that it reduces the error brought by the integral in the step length estimation and does not need to integrate the acceleration. PDR algorithm consists of three steps: step detection, step length calculation and heading detection. The previous position of the pedestrian can be used to calculat the current position:

(1) $$\left(\begin{array}{c} X_{k+1}\\ Y_{k+1} \end{array}\right)=\left(\begin{array}{c} X_{k}\\ Y_{k} \end{array}\right)+\left(\begin{array}{c} l\times \cos\varphi \\ l\times \sin\varphi \end{array}\right)$$

**Figure 3:**
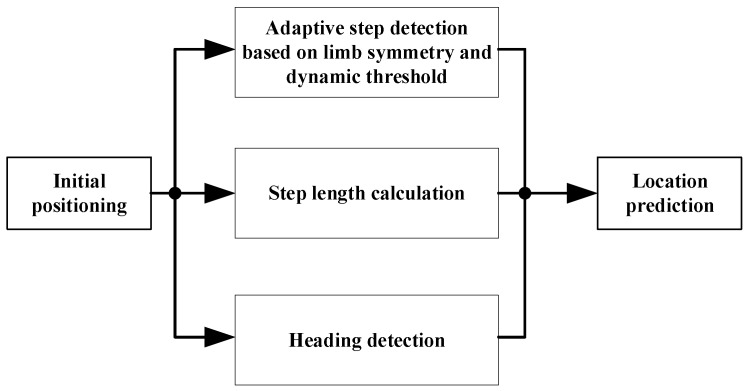
Improved PDR algorithm.

$\left(\begin{array}{c} X_{k+1}\\ Y_{k+1} \end{array}\right)$ and $\left(\begin{array}{c} X_{k}\\ Y_{k} \end{array}\right)$ respectively represent the current position and the previous position coordinates, respectively; l is the step length, and φ represents the heading value. The improved PDR algorithm proposed in this study is shown in Figure 3:

#### 2.2.1. Step Detection Method Based on Limb Symmetry and Dynamic Threshold

**Figure 4:**
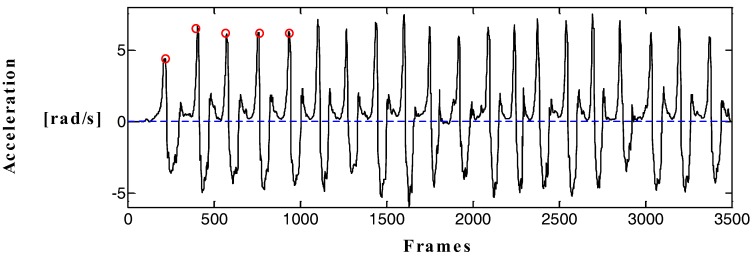
Acceleration of both feet (1 frame = 0.017 s).

The zero crossing method [14] and wave peak detection method [15] are two kinds of step detection methods. Owing to the presence of errors and the inevitable jitter in human motion, steps are misjudged by the traditional zero crossing method, making it difficult to detect the true steps accurately, as shown in Figure 4. A constant threshold value is set in the wave peak detection algorithm. The steps will be counted when the acceleration exceeds the threshold. Figure 4 illustrates that the human body is not in an absolutely uniform state during the course of motion, and the peak acceleration of each complete gait range is not fixed. Therefore, the peak detection method cannot accurately calculate the true number of steps. In view of the shortcomings of the traditional step detection method, this study proposes a new step detection method based on human motion characteristics and dynamic threshold to improve the accuracy of step detection. The inertial data in the process of walking show that the angular velocity of the left and right feet presents a certain periodicity and symmetry, and the features of angular velocity curves around the Y-axis is particularly evident. Therefore, to increase the accuracy of step detection, the traditional step detection method is improved based on the characteristics of the angular velocity of the Y-axis.

**Figure 5:**
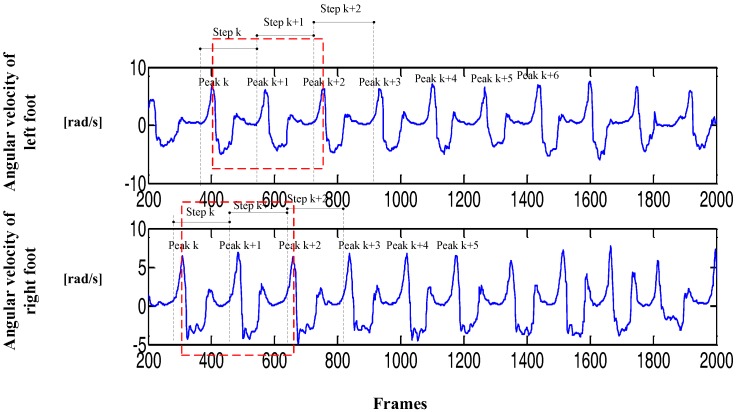
Amplitude variation of angular velocity in walking (1 frame = 0.017 s).

Figure 5 shows the change in angular velocity amplitude of the Y-axis. Step detection threshold should be constantly changing and continuous because walking is continuous. At the same time, the threshold of the current gait range is set according to the amplitude of the angular velocity curve of the previous three steps. The dynamic threshold can be calculated using Equations (2)–(4). In Equation (4), ${\bar{v}}_{peak}$ is the mean magnitude of the angular velocity of the previous three steps, and $v_{peak}^{\left(i\right)},v_{peak}^{\left(i-1\right)},v_{peak}^{\left(i-2\right)}$ are the peaks of the *i*, *i* + 1, and *i* + 2 steps, respectively.

(2) $${\bar{v}}_{peak}=\frac{1}{3}(v_{peak}^{\left(i\right)}+v_{peak}^{\left(i-1\right)}+v_{peak}^{\left(i-2\right)})$$

(3) $$l=\frac{{\bar{v}}_{peak}}{v_{peak}^{\left(i\right)}}$$

(4) $$th_{i+1}=\{\begin{array}{cc} AV+0.5 & 0\leq th_{i}\times l<AV+2\\ th_{i}\times l & AV+0.5\leq th_{i}\times l<AV+2\\ AV+2 & AV+2<th_{i}\times l \end{array}$$

$th_{i}$ represents the angular velocity threshold of the current step, and $th_{i+1}$ is the angular velocity threshold of *i* + 1 step. In Equation (3), l represents the ratio of ${\bar{v}}_{peak}$ and the peak value of the angular velocity of *i* step. Equation (4) is the updating algorithm of the dynamic threshold, and AV = 4 rad/s represents the angular velocity. To eliminate the slight disturbance in the walking process, the step number is not accumulated when the angular velocity is lower than $th_{\min}=AV+0.5$; by contrast, to eliminate the larger disturbance in walking, the step number is not accumulated when the angular velocity is greater than $th_{\max}=AV+2$. Therefore, the upper and lower bounds of the threshold are $th_{\max}=AV+2$ and $th_{\min}=AV+0.5$.

Table 1 compares the proposed PDR algorithm’s step detection method and the traditional step detection method. In actual use, the result of the step detection method based on a single foot is bound to be erroneously estimated. In the proposed step detection method, the walking steps of the left and right feet are calculated initially by the step detection method based on dynamic threshold, and then the checking mechanism is introduced to judge the state of the other foot according to the motion state of one foot. This method can improve the precision of step detection and reduce the PDR positioning error to a certain extent.

#### 2.2.2. Establishment of the Model of Step Length and Frequency

In the DR motion model, the accuracy of step length calculation will directly affect the final accuracy of the positioning result. At present, numerous common step length models are divided into linear [16] and nonlinear [17] step length calculation models. The method based on step length and frequency model is used to calculate real-time step length. Step length is proportional to the pace during the walking process, as shown in Equations (5) and (6), where, $S_{WF}(i)$ is the step length of step i, WF(i) represents the step frequency of step i, $v_{WF}(i)$ is the step frequency noise, and a and b are the linear coefficients. The relationship between step length and step frequency can be determined when a and b are known:

(5) $$S_{WF}(i)=aWF(i)+b+v_{WF}(i)$$

(6) $$\sigma_{WF}{}^{2}=E[v_{WF}{\left(i\right)}^{2}]$$

a and b can be calculated by the undetermined coefficient method. Firstly, the walking steps and time of the experimenter will be recorded. Second, the average step length will be calculated using walking distance and step number, and step frequency will be calculated by the step number and walking time. Finally, a and b values can be calculated using the least squares method, as follows:

(7) $$\{\begin{array}{c} S_{WF}(1)=aWF(1)+b\\ S_{WF}(2)=aWF(2)+b\\ \cdots \\ S_{WF}(3)=aWF(3)+b \end{array}$$

where, i=1,2,⋯n represent the number of tests, and the upper form can be written as:

(8) $$\left(\begin{array}{c} S_{WF}(1)\\ S_{WF}(2)\\ \vdots \\ S_{WF}(n) \end{array}\right)=\left(\begin{array}{c} WF(1),1\\ WF(2),1\\ \vdots \\ WF(n),1 \end{array}\right)\left(\begin{array}{c} a\\ b \end{array}\right)$$

The upper formula is simplified as follows:

(9) M=Ak

where, $M=\left(\begin{array}{c} S_{WF}(1)\\ S_{WF}(2)\\ \vdots \\ S_{WF}(n) \end{array}\right)$, $A=\left(\begin{array}{c} WF(1),1\\ WF(2),1\\ \vdots \\ WF(n),1 \end{array}\right)$, $k=\left(\begin{array}{c} a\\ b \end{array}\right)$.

k can be calculated by the least squares method:

(10) $$k={\left(A^{T}A\right)}^{-1}(A^{T}M)$$

#### 2.2.3. Heading Detection

**Figure 6:**
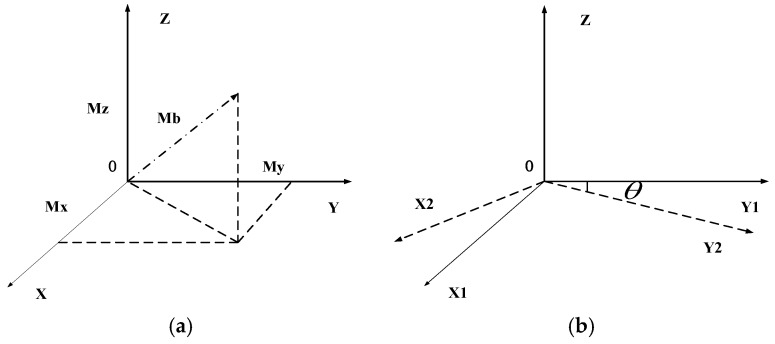
Geomagnetic vector component and angular velocity integration.

In the proposed PDR algorithm, the heading value is mainly calculated by magnetometer and gyroscope. In Figure 6a, the magnetometer output will determine the initial heading, and the change in direction will be determined by the gyroscope. The earth is a bipolar magnet. It has a component that always points to the magnetic north direction, and the projection of this component in three axes, as measured by magnetometer, can determine the direction of the carrier. The change in angle is determined by integrating the angular velocity values, as shown in Figure 6b.

**Figure 7:**
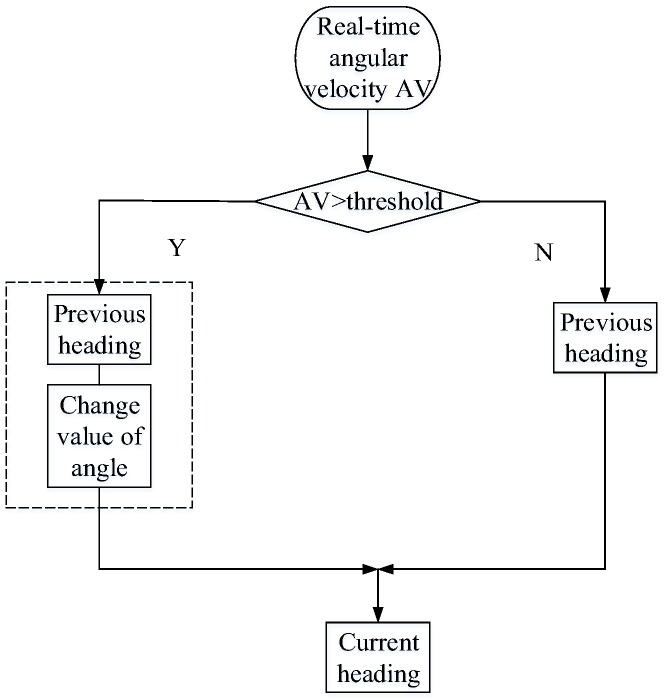
Flow chart of heading calculation.

To eliminate the noise caused by the movement of the leg jitter, the angular velocity threshold is set in the heading detection to determine whether the heading has changed effectively. In Figure 7, when the current angular velocity is greater than the threshold, the course is assumed to have changed, and the current heading is calculated by adding the previous course with the integral of the angular velocity. When the current angular velocity is less than the threshold, then the heading has not changed effectively, and the previous heading will be still used.

## 3. Data Fusion Strategy of Dynamic Pedestrian Positioning

Although the improved PDR method proposed in this study is more accurate than the traditional PDR algorithm in positioning results, it can still be difficult to utilize in the application requirements of some indoor environments because of the existence of cumulative error. The positioning accuracy of the UWB positioning method is relatively high, but its signal propagation is vulnerable to occlusion, and it cannot guarantee the continuous and stable positioning accuracy, causing its poor dynamic performance. The proper combination of the two methods can ensure high positioning accuracy and improve dynamic performance.

The position data of pedestrians are obtained by calculating using acceleration and angular velocity information obtained from inertial sensors through the PDR algorithm. Distance information is obtained by calculating the corresponding output data of the UWB module using time-of-flight (TOF) method. The location accuracy and dynamic performance of the indoor positioning system can be improved by fusing the two methods using the UKF filter.

**Figure 8:**
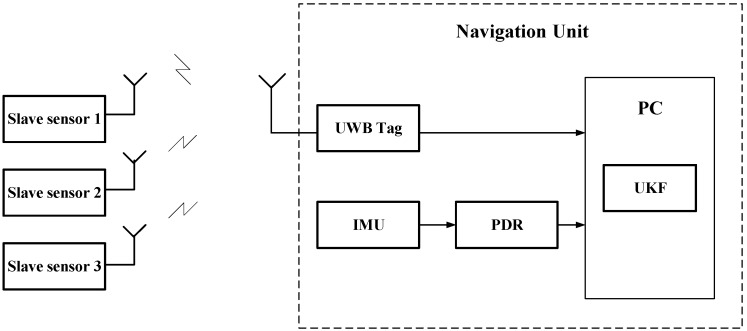
Structure diagram of the positioning system.

The indoor positioning system designed in this work consists of a hardware terminal and a navigation unit. The hardware includes the UWB sensors and IMU (Figure 8). The navigation unit of the PC estimates the position of the pedestrian by fusing the location data of UWB and the location and attitude calculated by IMU, thus plotting the pedestrian trajectory.

### 3.1. Data Fusion Strategy Based on UKF

**Figure 9:**
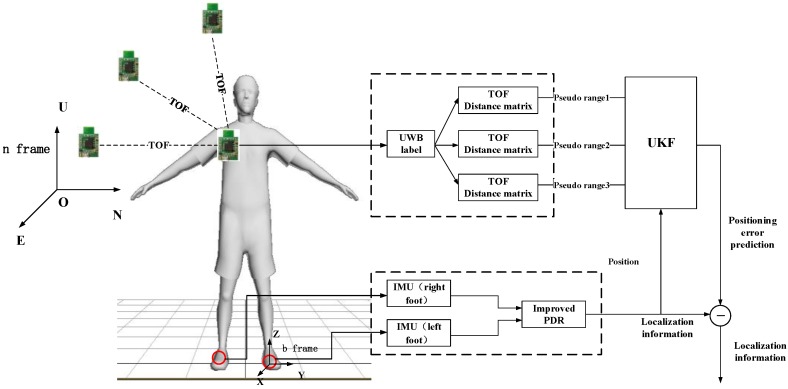
Data fusion strategy.

The basic architecture of the combined positioning system and the navigation coordinate system (n coordinate) and the carrier coordinate system (b coordinate) used in this work are shown in Figure 9. The integrated positioning system used by the algorithm is mainly composed of two parts: PDR and UWB positioning systems. Among these positioning systems, the IMU placed on the left foot and right foot obtains the pedestrian motion information through the built-in accelerometer, gyroscope and magnetometer. Then, the improved PDR algorithm is used to obtain the real-time position and travel trajectory of the pedestrian. The UWB positioning tag placed on the shoulder of a pedestrian calculates the distance between the positioning tag and the base station by measuring TOF data between them. On this basis, using UKF [18,19] is used to fuse the position information calculated by IMU and the pseudorange information measured by the UWB tag, so as to realize the accurate real-time indoor positioning of pedestrians.

### 3.2. Error Model of PDR/UWB Combined Positioning

The error model of the combined positioning system proposed in this work is a 15-dimensional state variable:

(11) $$\begin{array}{l} X=[\begin{array}{ccccccccc} \varphi_{E} & \varphi_{N} & \varphi_{U} & \delta v_{E} & \delta v_{N} & \delta v_{U} & \delta P_{E} & \delta P_{N} & \delta P_{U} \end{array}\\ \begin{array}{cccccccccc} & & & & \varepsilon_{bx} & \varepsilon_{by} & \varepsilon_{bz} & \nabla_{ax} & \nabla_{ay} & \nabla_{az} \end{array}] \end{array}$$

where, $\begin{array}{ccc} \varphi_{E} & \varphi_{N} & \varphi_{U} \end{array}$ represent the attitude angle errors in the east, north, and up directions; $\begin{array}{ccc} \delta v_{E} & \delta v_{N} & \delta v_{U} \end{array}$ represent the velocity errors in the east, north, and up directions; $\begin{array}{ccc} \delta P_{E} & \delta P_{N} & \delta P_{U} \end{array}$ are the position errors; $\begin{array}{ccc} \varepsilon_{bx} & \varepsilon_{by} & \varepsilon_{bz} \end{array}$ represent the gyroscope errors; $\begin{array}{ccc} \nabla_{ax} & \nabla_{ay} & \nabla_{az} \end{array}$ are the acceleration errors.

## 4. Experimentation

In this work, an improved PDR algorithm for pedestrian localization is proposed by improving the traditional PDR algorithm according to the symmetry of the human body. A complete indoor positioning system is designed by fusing PDR algorithm and UWB positioning method using UKF, which not only ensures the positioning accuracy under static conditions, but also improves the dynamic performance of the positioning system effectively. To verify the effectiveness of the indoor positioning system, first, the localization accuracy of the improved PDR algorithm and the traditional PDR algorithm is compared and the error of the two methods is analyzed. Experiments are then designed to verify the static positioning accuracy and dynamic performance of the proposed method. In the static verification, the positioning results of the improved PDR positioning, UWB positioning and UKF fusion positioning are analyzed, and the positioning accuracy and positioning error of the three methods are compared. In the dynamic test, a dynamic positioning reference system is designed based on capacitive touch sensing, the positioning system and the verification platform are set to clock synchronization, the positioning accuracy of each time is analyzed, and the error curve is plotted.

### 4.1. Comparison of Positioning Results between Improved PDR and Traditional PDR

**Figure 10:**
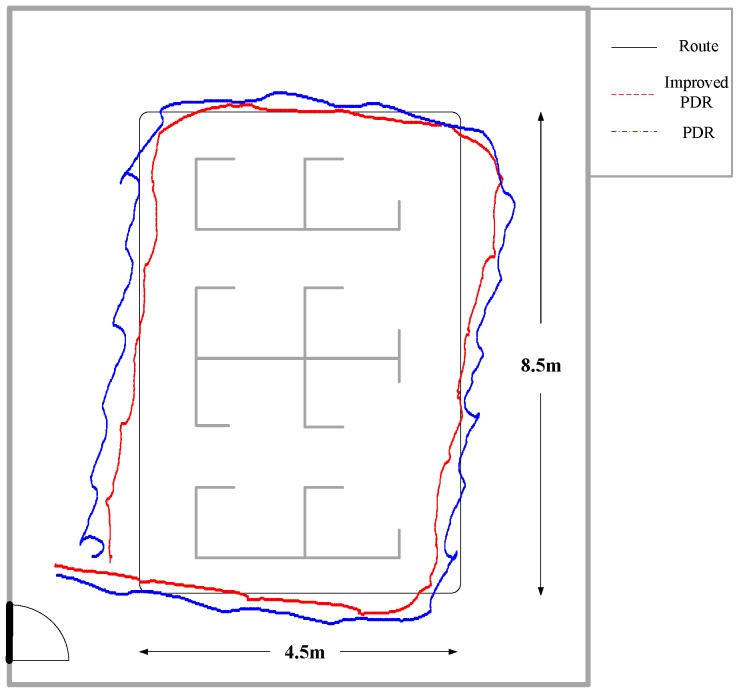
Location results of traditional PDR and improved PDR.

Figure 10 shows the comparison of localization results between the traditional PDR and the improved PDR algorithm in this paper. The test site is a laboratory 11.7 m long and 7.5 m wide. The black curve in the figure represents the walking trajectory, which is a rectangle 8.5 m long and 4.5 m wide. The blue curve represents the traditional PDR trajectory, and the red curve is the predictive trajectory of the improved PDR algorithm proposed in this work. The positioning accuracy of this method is improved by 20% compared with the traditional PDR algorithm, an improvement that can provide the positioning accuracy of approximately 51.25 cm, as shown in Table 2.

### 4.2. Static Verification

**Figure 11:**
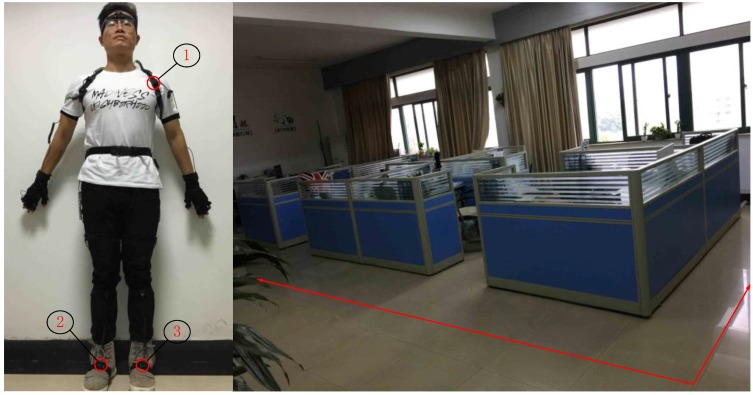
Node arrangement and experimental site.

**Figure 12:**
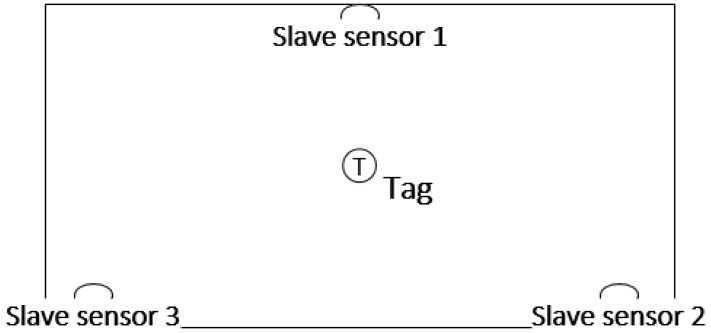
Outlet of UWB sensors.

To verify the performance of the proposed indoor positioning method, the laboratory (Figure 11) is chosen as the experimental site. Table 3 shows the node arrangement. The walking trajectory is a rectangle 8.5 m long and 4.5 m wide, as represented by the red line. Figure 12 shows the layout of the UWB base station, UWB sensors are important parts of UWB positioning, as they receive the signal and provide the time and angle data required, as well as play a role in achieving the communication between the tag and the positioning platform. Therefore, the number and layout of sensors will play a decisive role for positioning results. A test platform using three base stations and one tab can cover the entire laboratory and meet the requirements of positioning. In addition, the UWB tab is at least 15 cm away from the wall, table and other obstacles. Otherwise, the location data will be inaccurate. The base station is placed 3 ft, more than 2 m, above the ground. The sensors can measure the TOF of the UWB pulses transmitted by the tags, therefore providing a centralized estimate of the tag positions.

**Figure 13:**
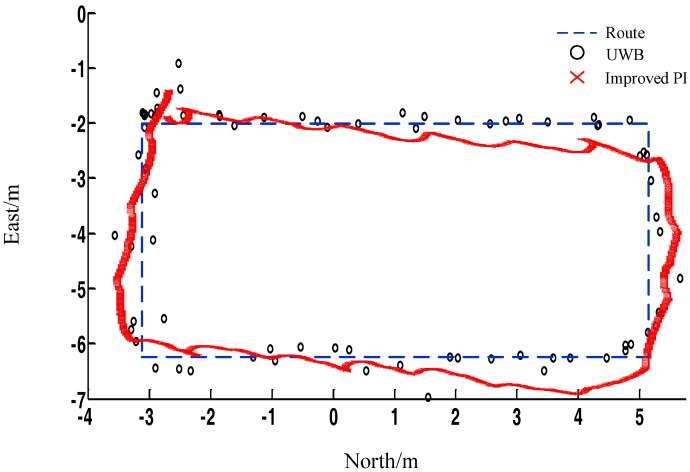
Trajectories of UWB and improved PDR.

In Figure 13, the blue dotted line represents the actual walking trajectory of the pedestrian in the experiment, and the black circle indicates the location data computed by UWB positioning, and the red fork represents the location data calculated by PDR. Based on the data shown in the figure, UWB positioning can provide higher positioning accuracy compared with PDR positioning. However, an obvious delay in the position calculation of UWB and a signal loss at the same time are observed, which cannot provide continuous positioning results. PDR positioning can provide continuous positioning data, but the accuracy cannot meet the requirements of indoor positioning.

**Figure 14:**
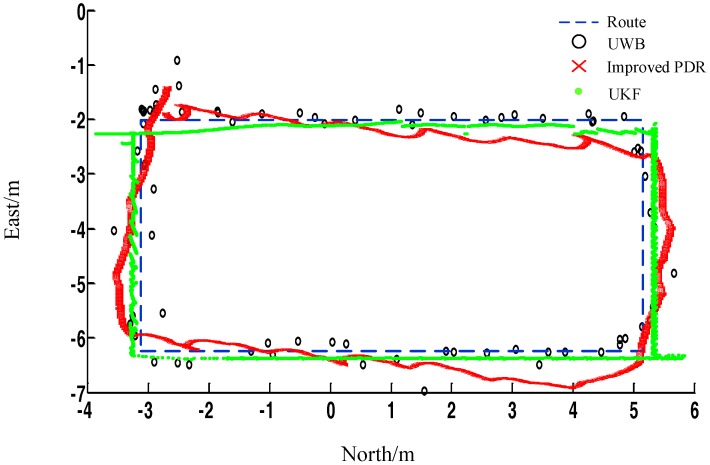
Positioning trajectory through UKF filter.

In Figure 14, the solution trajectory after UKF fusion is represented by green points with a positioning accuracy of 10–15 cm. At the same time, it can be seen that the dynamic performance is greatly improved compared with the single UWB. The specific dynamic verification is introduced in the next section.

The error analysis of the three methods is shown in Table 4:

As shown in Table 4, the results of static verification show that the proposed method can effectively improve the positioning accuracy under static positioning with an average positioning error of 0.13 m.

To verify the high accuracy performance of the proposed method under static positioning, experiments have been designed to compare the positioning results of the proposed method and the latest localization methods. In [20], the author proposed an approach based on support vector regression to estimate the received signal strength at non-site-surveyed positions of the environment. The proposed method could be used to improve the resolution of fingerprint-based indoor WiFi localization systems without increasing the site survey effort. In [21], a new Wi-Fi based indoor localization technique is proposed that achieves significantly improvement of indoor positioning accuracy with the help of Li-Fi assisted coefficient calibration. The proposed technique leverages indoor existing Li-Fi lighting and Wi-Fi infrastructure, and results in a cost-effective and user-convenient indoor accurate localization framework. In this work, experimental study and measurements are conducted to verify the performance of the proposed idea. Experimental results in this work demonstrate an accuracy improvement of 80% compared with existing WiFi based positioning systems.

**Figure 15:**
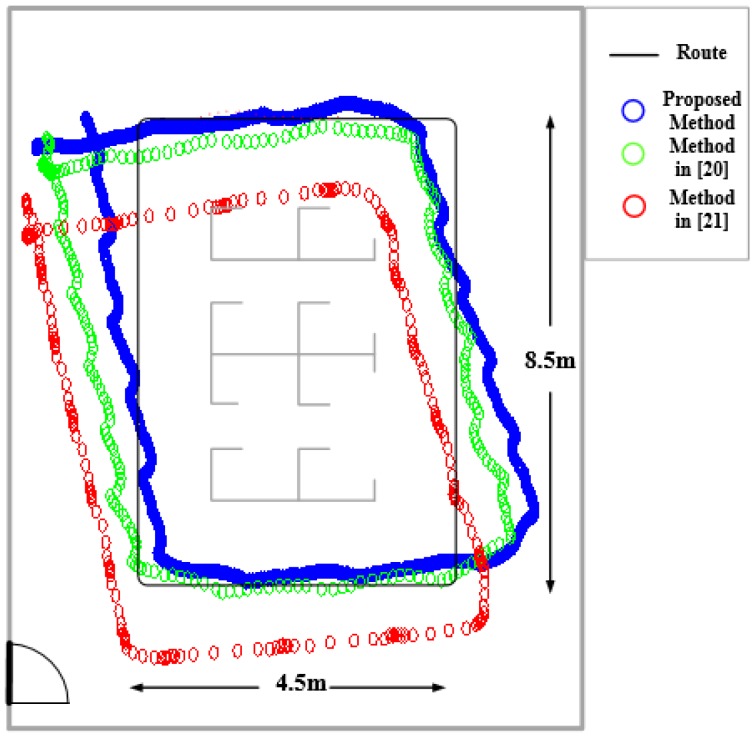
Comparison of positioning results between the proposed method and methods in [20,21].

As shown in Figure 15, the method in the current work has better static positioning performance than methods in [20,21]. Table 5 further proves the validity of the proposed method. In addition, Figure 15 confirms that the method has better dynamic performance.

### 4.3. Dynamic Verification

The dynamic positioning verification platform verifies the accuracy and real-time performance of the indoor localization method. Therefore, the reference system must be able to accurately locate the monitoring points and give reference results quickly. The positioning reference system used in this work is based on the principle of capacitive touch induction. The maximum dynamic positioning error is 6 cm, and the dynamic response time is approximately 16 ms, which can meet the requirements of the positioning reference system.

#### 4.3.1. Design of the Verification Platform

**Figure 16:**
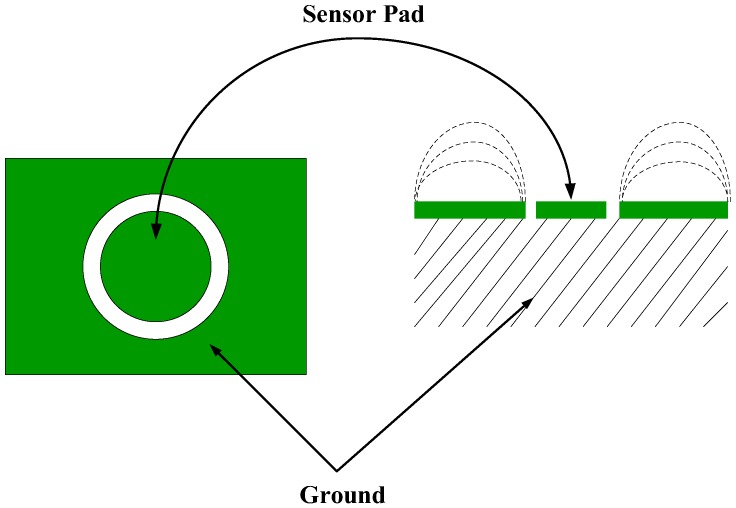
Capacitance induction principle.

The indoor positioning dynamic reference system used in this work is based on the principle of capacitive touch induction, Figure 16 shows a capacitive touch panel, where the middle green round is copper, which can be called a “key”. These buttons lead to a wire attached to the MCU, which detects whether the button is “pressed” by a wire. Figure 16 shows the capacitance sensing module designed in this work. The module is mainly composed of a capacitance induction chip MPR121 and a master chip STM32F103C8T6, and the capacitor induction chip and MCU communicate by IIC. Each capacitive sensing module contains six capacitive sensing chips, each spaced at 6 cm. To apply the capacitive sensing module to pedestrian location detection, the circular copper strip is designed as a long strip shape. In the walking process, when the foot touches the capacitor module, the corresponding capacitance sensing chip will react immediately and output in real-time the pedestrian position. The low cost and fast response of the MPR121 chip provide the precondition of the validity of the positioning reference system.

**Figure 17:**
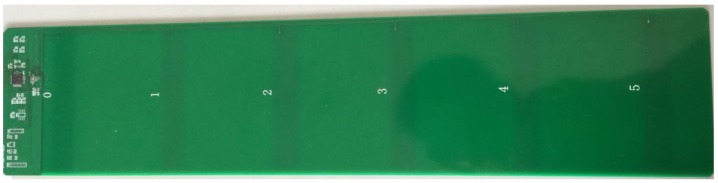
Hardware of capacitor module.

**Figure 18:**
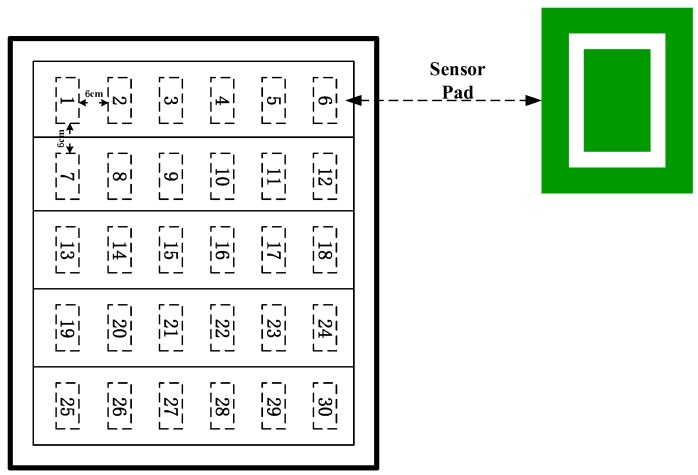
Outlet of capacitor module.

Figure 17 shows the capacitive sensing module:

In the test, the capacitive sensing module is arranged in a grid format, and each module is connected by a CAN bus (Figure 18). Each capacitor chip has its own number and marks the coordinates of each chip. When the pedestrian walks in the area, the reference system can quickly locate the coordinates of the pedestrian. When the pedestrian passes through the capacitance induction module, the capacitance sensing chip quickly senses the foot action, and calculates the location of the pedestrian according to the positioning algorithm. The distance between each capacitor sensing chip determines the positioning accuracy of the positioning reference system. In this work, a spacing of 6 cm is selected, and the positioning accuracy can meet the precision requirements of the positioning reference system. At the same time, the module keeps the clock synchronized with the indoor positioning system, and the dynamic coordinates calculated by the capacitance induction module and the proposed indoor positioning are compared to verify the performance of the dynamic positioning method.

**Figure 19:**
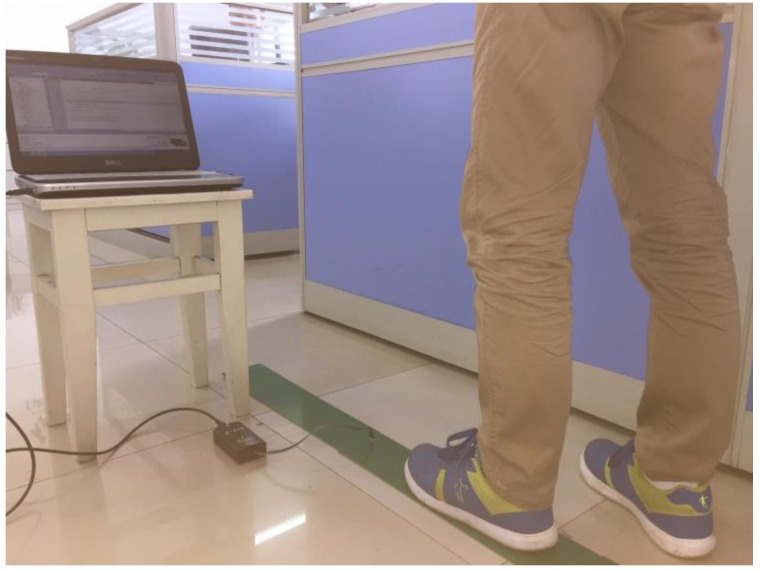
Outlet of capacitor module.

Figure 19 shows the actual use of the capacitor module in the experiment. The capacitance sensing chip quickly senses the foot action, and the navigation unit of the PC shows the walking trajectory.

#### 4.3.2. Calculating the Dynamic Response Performance of the Dynamic Positioning Reference System

To verify the real-time performance of this method, the performance of dynamic response, which is also an index, should not be ignored by the location verification system. The dynamic response time of the reference system based on the principle of capacitive touch induction is mainly composed of four parts: ① Chip induction time, ② IIC communication time, ③ Algorithm running time, ④ CAN bus communication time, all of which are expressed in the following formula:

(12) $$t_{D}=t_{R}+t_{I}+t_{A}+t_{C}$$

In the formula, $t_{D}$ represents the dynamic response time; $t_{R}$ represents the chip induction time, which is 16 ms; $t_{I}$ represents the IIC communication time, approximately 4 μs; $t_{A}$ represents the algorithm running time, approximately 10 ns; and $t_{C}$ represents the CAN bus communication time, approximately 4 μs. The dynamic response time of the reference system is approximately 16 ms calculated by the formula.

### 4.4. Verification Result

In daily activities, the speed of the human body is constantly changing. Therefore, the dynamic positioning performance under different moving speeds needs to be analyzed to verify the dynamic performance of the positioning method. The dynamic verification test was carried out in three groups, and the results were compared with a pedestrian under normal walking speed, jogging and fast running speeds. The speed of movement was 1, 2 and 3 m/s in the straight line movement.

**Figure 20:**
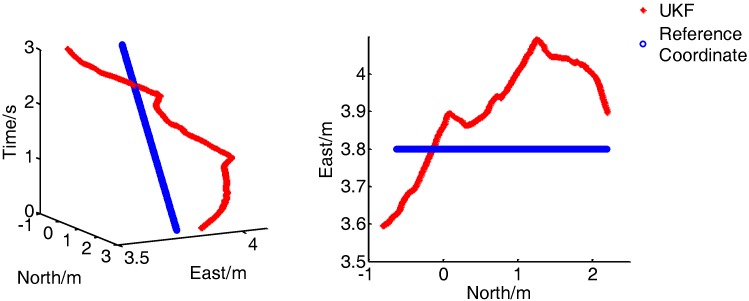
Results for dynamic tracking of position (1 m/s).

(1) Dynamic verification under walking speed (1 m/s):

Figure 20 presents the comparison of the real-time position between the combined positioning system proposed and the reference system when the walking speed is 1 m/s. In the left figure, the X and Y axes represent the position of the pedestrian, and the Z axis represents the time. As shown in the diagram, under the normal walking speed, the integrated navigation system can continuously and stably output the pedestrian position, and has excellent dynamic performance and high positioning accuracy.

**Figure 21:**
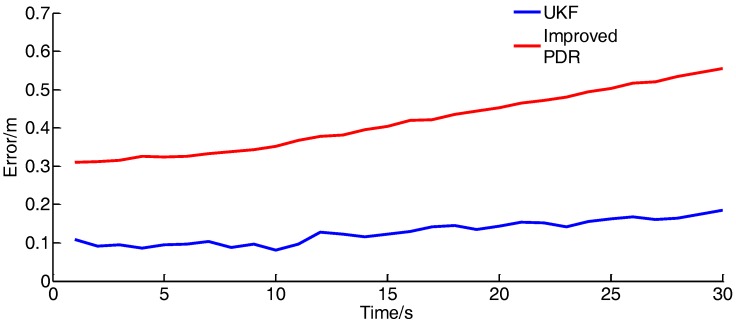
Location error curve. (1 m/s).

To verify the positioning accuracy of the method, the positioning error of the integrated positioning method and the improved PDR method are compared under the dynamic positioning, as shown in Figure 21: 

As shown in the diagram, the positioning error of the single PDR method will diverge with time, and the positioning accuracy of the combined method is stable owing to the correction of UWB.

(2) Dynamic verification under jogging speed (2 m/s)

**Figure 22:**
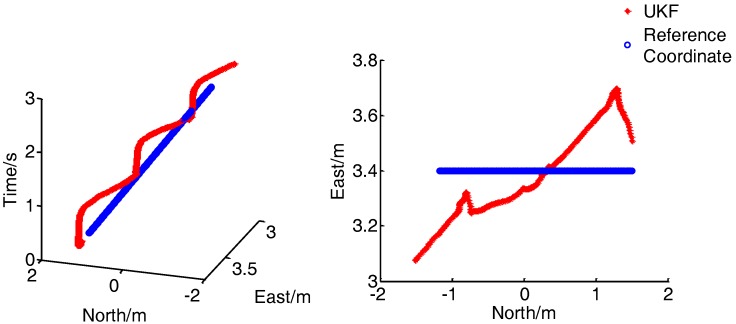
Results for the dynamic tracking of position (2 m/s).

**Figure 23:**
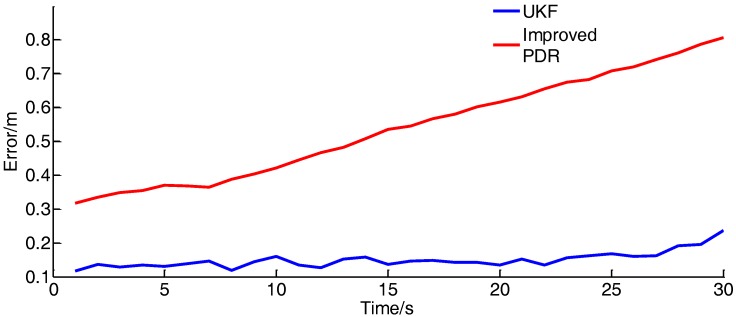
Location error curve (2 m/s).

**Figure 24:**
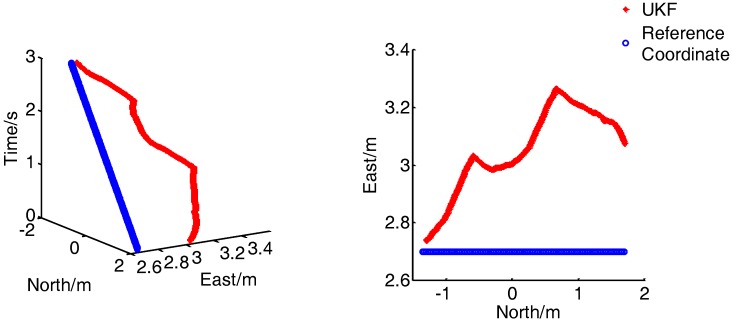
Results for the dynamic tracking of position (3 m/s).

**Figure 25:**
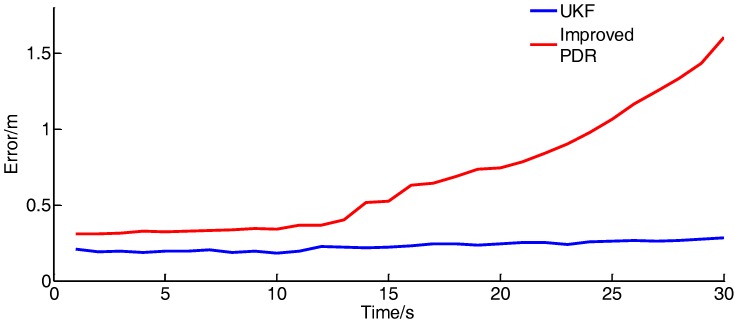
Location error curve (3 m/s).

As shown in Figure 22, the method used in this work still has good dynamic performance under the motion of 2 m/s, and the positioning accuracy is slightly inferior to the positioning accuracy at walking speed. The error curve is shown in Figure 23:

(3) Dynamic verification under running speed (3 m/s):

As shown in Figure 24, the dynamic performance of the method can meet the requirements of the human body in the running situation, but, the positioning accuracy is lower than that when walking and jogging, as shown in Figure 25.

The average error analysis of the integrated positioning system and the modified PDR dynamic verification test is shown in Table 6:

The experimental results show that the designed indoor positioning system can meet the dynamic performance of the human body at normal speed, but the positioning accuracy is different at different speeds. The location error increases gradually with the increase of speed, but the error will not diverge over time because of the error correction of UWB.

## 5. Conclusions

In this work, an integrated positioning method of UWB and an improved PDR is proposed, and the advantages and disadvantages of UWB and PDR are analyzed respectively. According to the symmetry of the human body in motion, the traditional PDR algorithm is improved, and the location data of two methods are fused to achieve complementary advantages, to meet the positioning requirements in complex indoor environments. As UWB cannot complete the positioning task when the signal is blocked, PDR is used to make up for these defects. At the same time, UWB can effectively eliminate the accumulated position, velocity and attitude error of the PDR algorithm. The experimental results show that the positioning error of this method is reduced by 74.5% and 43.5% compared to that of PDR and UWB respectively. The average accuracy of the proposed method can reach 10–15 cm under both dynamic and static conditions.

## Figures and Tables

**Table 1:** Results of step detection.

	Ground Truth User 1	User 1 with PDR	User 1 with Improved PDR	Ground Truth User 2	User 2 with PDR	User 1 with Improved PDR
Number of steps	200	181	193	300	274	291

**Table 2:** Error analysis of PDR and improved PDR.

	Improved PDR	PDR
Maximum error/m	0.95	1.23
Average error/m	0.51	0.93
Minimum error/m	0.32	0.35

**Table 3:** Node arrangement.

Number	Hardware Module
1	UWB Tab
2	IMU(R)
3	IMU(L)

**Table 4:** Error analysis of three methods.

	Improved PDR	UWB	UWB/Improved PDR
Maximum error/m	0.95	0.54	0.33
Average error/m	0.51	0.23	0.13
Minimum error/m	0.32	0.12	0.02

**Table 5:** Error analysis of three methods.

	Method in [21]	Method in [20]	UWB/Improved PDR
Maximum error/m	1.78	0.72	0.35
Average error/m	1.24	0.46	0.15
Minimum error/m	0.22	0.13	0.05

**Table 6:** Error analysis of dynamic experiment.

Speed/(m/s)	Average Error of UKF/(m)	Average Error of Improved PDR/(m)
1	0.129706	0.504338
2	0.155324	0.597811
3	0.195338	0.80149

## Abbreviations

The following abbreviations are used in this manuscript:

UWB: ultra-wide band
UKF: unscented Kalman filter
PDR: pedestrian dead reckoning
IMU: inertial components
ZUPT: zero-velocity update
